# HBx-induced MiR-1269b in NF-κB dependent manner upregulates cell division cycle 40 homolog (CDC40) to promote proliferation and migration in hepatoma cells

**DOI:** 10.1186/s12967-016-0949-y

**Published:** 2016-06-27

**Authors:** Xiao-xiao Kong, Yan-ru Lv, Li-ping Shao, Xiang-yang Nong, Guang-ling Zhang, Yi Zhang, Hong-xia Fan, Min Liu, Xin Li, Hua Tang

**Affiliations:** Tianjin Life Science Research Center, School of Basic Medical Sciences, Tianjin Medical University, 22 Qi-Xiang-Tai Road, Tianjin, 300070 China; The People’s Hospital of Guangxi Zhuang Autonomous Region, Nanning, Guangxi Zhuang Autonomous Region China; Tangshan Key Laboratory for Preclinical and Basic Research on Chronic Diseases, School of Basic Medical Sciences, North China University of Science and Technology, Tangshan City, Hebei Province China

**Keywords:** HCC, HBx, NF-κB, miR-1269b, CDC40

## Abstract

**Background:**

Occurrence and progression of hepatocellular carcinoma (HCC) are associated with hepatitis B virus (HBV) infection. miR-1269b is up-regulated in HCC cells and tissues. However, the regulation of miR-1269b expression by HBV and the mechanism underlying the oncogenic activity of miR-1269b in HCC are unclear.

**Methods:**

Reverse transcription quantitative PCR (RT-qPCR) was used to measure the expression of miR-1269b and target genes in HCC tissues and cell lines. Western blot analysis was used to assess the expression of miR-1269b target genes and related proteins. Using luciferase reporter assays and EMSA, we identified the factors regulating the transcriptional level of miR-1269b. Colony formation, flow cytometry and cell migration assays were performed to evaluate the phenotypic changes caused by miR-1269b and its target in HCC cells.

**Results:**

We demonstrated that the expression levels of pre-miR-1269b and miR-1269b in HBV-positive HepG2.2.15 cells were dramatically increased compared with HBV-negative HepG2 cells. HBx was shown to facilitate translocation of NF-κB from the cytoplasm to the nucleus, and NF-κB binds to the promoter of miR-1269b to enhance its transcription. miR-1269b targets and up-regulates CDC40, a cell division cycle 40 homolog. CDC40 increases cell cycle progression, cell proliferation and migration. Rescue experiment indicated that CDC40 promotes malignancy induced by miR-1269b in HCC cells.

**Conclusion:**

We found that HBx activates NF-κB to promote the expression of miR1269b, which augments CDC40 expression, contributing to malignancy in HCC. Our findings provide insights into the mechanisms underlying HBV-induced hepatocarcinogenesis.

## Background

HCC is the second most common causes of cancer death in the world, with 0.8 million deaths per year worldwide [1]. Chronic HBV infection is closely associated with the initiation and development of HCC. Approximately 50 % of all HCC cases are linked to HBV chronic infection globally [2]. The mechanisms of HBV-induced malignant transformation are not completely clear, however, accumulated evidence has indicated that the HBV X protein acts as a multifunctional regulator in carcinogenesis of HCC [3, 4]. HBx is the smallest protein (17 kDa) encoded by HBV [5]. Numerous studies have shown that HBx plays a crucial role in the development of HBV-related HCC. The HBx protein promotes cellular proliferation and invasion through several mechanisms [6–8]. Among its various roles as tumor promoter, HBx has been confirmed to function as a transcriptional activator. The HBx protein does not bind to DNA but interacts directly with several transcription factors to either suppress or activate them, for example, repression of p53 [9, 10] and activation of C/EBP [11], AP-1 [12], AP-2 [13], and STAT3 [14]. The transcription factor NF-κB was identified as the first HBx-responsive motif that could be activated by HBx [13]. Sequential reports demonstrated that HBx modulates the expression of various NF-κB target genes [14–16]. However, the molecular mechanisms of HBx-induced malignancy in HCC are complicated and far from being fully understood.

The NF-κB transcription factor belongs to the proto-oncogene family Rel, which is composed of five proteins, p50 (NF-κB1), p52 (NF-κB2), RelA (p65), RelB and c-Rel (Rel). Two of these proteins can form a homodimeric or heterodimeric transcription factor complex, and all of them possess DNA binding and protein dimerization activity because of their N-terminal domain. Known as the Rel homology (RH) domain [17]. Except for the NF-κB p65/c-Rel heterodimer, the NF-κB dimers bind to specific DNA elements with a consensus sequence of 5′-GGGRNYYYCC-3′ (R-unspecified purine; N-any base; Y-unspecified pyrimidine) called the κB sites. NF-κB usually refers to the p50/p65 heterodimer because of its high expression levels in somatic cells, and it binds various κB sites. NF-κB needs to be translocated from the cytoplasm to the nucleus by an activator, for example, tumour necrosis factor alpha (TNF-α) can be an activator of NF-κB and positively regulates NF-κB import into the nucleus.

MicroRNAs (miRNAs) are small non-coding RNAs that are highly conserved and can up- or down-regulate the expression of their target genes [18, 19]. miRNAs may function as tumor suppressors or oncogenes in various cancers [20]. miR-1269 has been reported to be an oncogene in HCC cells, and its expression level is substantially higher in HCC tissues than in non-cancerous liver tissues [21, 22]. miR-1269 is also known as miR-1269a or miR-1269b, which share the same mature sequence, but they are derived from chromosomes 4 and 17 respectively. Whether HBV infection influences the expression of miR-1269a or miR-1269b is unknown.

In this study, we found that the HBx protein activates miR-1269b expression by facilitating the nucleus translocation of NF-κB from the cytoplasm in HCC. The precursor of miR-1269a and miR-1269b was detected in HepG2 and HepG2.2.15 cells by reverse transcription qPCR (RT-qPCR). Mature miR-1269 and pre-miR-1269b, but not the pre-miR-1269a, were highly expressed in HepG2.2.15 cells. Thus, up-regulated miR-1269b and pre-miR-1269b are related to HBV. Furthermore, we found that miR-1269b targets and enhances CDC40 expression, a splicing factor involved in cell cycle control [23, 24], which mediates the effects of miR-1269b on cell growth, cell cycle and cell migration in HCC cells. These findings reveal a new pathway of HBx/NF-κB/miR-1269b/CDC40 that contributes to tumorigenesis in HBV-associated HCC.

## Methods

### Cell lines, cell cultures and cell transfection

The human hepatocarcinoma cell lines HepG2 and SMMC-7721 (HBV-negative) were purchased from the American Type Culture Collection. HepG2.2.15 (HBV-positive) cells were obtained from the Shanghai Second Military Medical University. HepG2 and HepG2.2.15 cells were cultured in MEM-α (HyClone, China) containing 10 % FBS (Gibco, USA), 100 U/ml penicillin and streptomycin, and 5 mmol/l glutamine. SMMC-7721 was cultured in RPMI-1640 (HyClone, China) supplemented with 100 U/ml penicillin and streptomycin. Cells were incubated in a humidified 37 °C incubator with 5 % CO_2_.

Cell transfection was performed using the Lipofectamine 2000 Reagent (Invitrogen, Carlsbad, CA, USA) according to the manufacturer’s instructions.

### Plasmid construction

A pri-miR-1269b PCR product (see primers below) was inserted into the pcDNA3 vector between its BamHI and EcoRI sites, and 2′-*O*-methyl-modified antisense oligonucleotides for miR-1269b (ASO-miR-1269b) and scramble control oligonucleotides (ASO-NC) were purchased from the GenePharma (Shanghai, China). The promoter of pri-miR-1269b, which contained the predicted NFκB1 binding sites (pMiR-1269b-luc), and a promoter of pri-miR-1269b from which the predicted NFκB1 binding sites were removed (pMiR-1269b-luc-mut) were cloned into the XholI-EcoRI and KpnI-EcoRI sites of the pGL3-Basic-luc^+^ vector, respectively. The CDC40 gene was amplified from a cDNA derived from HepG2 cells and cloned into the pcDNA3/HA tag vector between its EcoRI and XhoI sites. CDC40 knockdown primers were synthesized from GenePharma (Shanghai, China). The two strands were annealed and then cloned into the BamHI and HindIII sites of the pSilencer2.1-neo vector (Ambion, Austin, TX). The fragments of CDC40 3′UTR containing the miR-1269b binding site and its mutated form were cloned into pcDNA3-EGFP vectors between the BamHI and EcoRI sites. The sequences of all primers and oligos are shown in Table 1.

**Table 1:** Primers used in plasmid construction

Name	Primer sequence
pMiR-1269b-luc sense	5′-GACAACTCGAGTGCCCTCACCTTGCCC-3′
pMiR-1269b-luc anti-sense	5′-GACCGAATTCAAATGTCAAAGAACCTGG-3′
pMiR-1269b-luc-mut sense	5′-GGCCAGGTACCTGCCCTCACCTTGCCC-3′
pMiR-1269b-luc-mut anti-sense	5′-AGCGAATTCCAAACCAAGGAGCAAGTCCG-3′
pre-miR-1269b sense	5′-CGCGGATCCACTGAGTTTCGGGTTCTG-3′
pre-miR-1269b anti-sense	5′ -CGGAATTCGTTGGCTCACATAATCACA-3′
ASO-miR-1269b	5′-CCAGUAGCAUGGCUCAGUCCAG-3′
ASO-NC	5′-CAGUACUUUUGUGUAGUACAA-3′
CDC40 sense	5′-GAAGAATTCATGTCGGCTGCGATTGCAGCTCTGGC-3′
CDC40 anti-sense	5′-GAAGACTCGAGCGATCCCACAATTTAATGAGACCATCCCAAC-3′
CDC40-shR-top	5′-GATCCGTGGTTGGGATGGTCTCATTACTCGAGTAATGAGACCATCCCAACCACTTTTTGA-3′
CDC40-shR-bottom	5′-AGCTTCAAAAAGTGGTTGGGATGGTCTCATTACTCGAGTAATGAGACCATCCCAACCACG-3′
CDC40-3′UTR sense	5′-GATCCAAGTACGAGGGGTAGAGTCCAAGATGTCAACTTGTGGTACCG-3′
CDC40-3′UTR anti-sense	5′-AATTCGGTACCACAAGTTGACATCTTGGACTCTACCCCTCGTACTTG-3′
CDC40-3′UTRmut sense	5′-GATCCAAGTACGAGGGGTAGACTGGAAGATGTCAACTTGTGGTACCG-3′
CDC40-3′UTRmut anti-sense	5′-AATTCGGTACCACAAGTTGACATCTTCCAGTCTACCCCTCGTACTTG-3′

### RNA extraction and reverse transcription quantitative PCR (RT-qPCR)

Total RNAs were extracted from HepG2 and SMMC-7721 cells by using TRIzol reagent (Invitrogen, Carlsbad, CA). RT-qPCR assays were performed as described in a previous study [25]. The primers used for RT-q PCR are listed in Table 2.

**Table 2:** Primers used in qRT-PCR

Name	Primer sequence
U6 RT primer	5′-GTCGTATCCAGTGCAGGGTCCGAGGTATTCGCACTGGATACGACAAAATATGGAAC-3′
miR-1269b RT primer	5′-GTCGTATCCAGTGCAGGGTCCGAGGTGCACTGGATACGACCCAGTAGC-3′
U6 forward	5′-TGCGGGTGCTCGCTTCGGCAGC-3′
U6 reverse	5′-CCAGTGCAGGGTCCGAGGT-3′
miR-1269b forward	5′- TGCGCTGGACTGAGCCATGC-3′
pre-miR-1269b-qPCR-S	5′-CAGGCTGGGAGAAAGACC-3′
pre-miR-1269b-qPCR-AS	5′-GGAAGTTGGCTCACATAATC-3′
pre-miR-1269a-qPCR-S	5′ TGGATTGCCTAGACCAGGG 3′
pre-miR-1269a-qPCR-AS	5′ GCTGGAGACCAGGGAAGCCAG 3′
CDC40-qPCR-S	5′-GTATGCTCCTTGCCCTTACGG-3′
CDC40-qPCR-AS	5′-AATGGCAGCAGCTAGGCAAT-3′
β-actin-qPCR-S	5′-CGTGACATTAAGGAGAAGCTG-3′
β-actin-qPCR-AS	5′-CTAGAAGCATTTGCGGTGGAC-3′

### Luciferase reporter assay

SMMC-7721 cells were seeded into 48-well culture plates. Three wells were seeded per group. A total of 500 ng of pGL3-Ctrl-luc^+^ vector, 500 ng of pMiR-1269b-luc or 500 ng of pMiR-1269b-luc-mut and 50 ng of the pRL-TK plasmid were co-transfected into the cells of each well for 24 h. A Dual-Luciferase Reporter Assay System (Promega, Madison, WI) was used to analyze the experimental results. The rest of the luciferase activity tests were cultured in the same way for 24 h, the cells were treated with or without TNF-alpha.

### Electrophoretic mobility shift assay (EMSA)

EMSA was performed using a LightShift Chemiluminescent EMSA Kit (Thermo) according to the manufacturer’s protocol. The 3′-end-biotin-labeled double-stranded DNA and the unlabeled DNA (dsDNA) oligonucleotide probes or competitors (GenePharma, Shanghai, China) containing an NF-κB-binding site corresponded to the sequence 5′-GCCAGACCCGGGACCCTCCTTTTGAGGC AC-3′ (probe 1) or 5′-TAGCTGAGAGGGTGTTTCCAAAAGA GACCA-3′ (probe 2). DNA binding complexes were formed in 20-μl samples containing 20 fmol biotin-labeled oligonucleotides, 10 μg nuclear extracts, 2 μl 10 × binding buffer, 1 μl 50 % glycerol, 5 mM MgCl_2_, and 0.05 % NP-40. For competitor EMSA, 20- or 100-fold dilutions of unlabeled oligonucleotides were added prior to the addition of the labeled probe. The samples were run on 6 % non-denaturing polyacrylamide gels with 0.5×TBE buffer and then transferred to a nylon membrane at 380 mA for 40 min. The membranes were crosslinked at 120 mJ/cm^2^ using a commercial UV-light apparatus for 5 min. A chemiluminescent detection method was performed where in a luminal/enhancer solution and a stable peroxide solution were used as described by the manufacturer.

### Western blot analysis

Total protein was extracted from 24 to 48 h post-transfected cells in RIPA lysis buffer. Isolated proteins were separated using 10 or 12 % SDS-PAGE and then electroblotted onto PVDF membranes. The primary antibodies used in this study included NF-κB, CENPA, E-cadherin, vimentin and GAPDH, which were obtained from Saier Co. (Tianjin, China). The CDC40 antibody and the secondary goat anti-rabbit antibody were obtained from Sigma and used according to standard protocols.

### Fluorescence assay

HepG2 and SMMC-7721 cells were cultured in 24-well culture plates at a density of 1 × 10^4^ or 3 × 10^4^ cells per well in MEM-α or RPMI-1640 medium containing 10 % fetal bovine serum (FBS). Cells were cultured at 37 °C for 24 h before transfection. Cells were co-transfected with the EGFP reporter constructs and miRNA expression construct. After 48 h incubation, the cells were collected and lysed in RIPA buffer. The RFP expression vector pDsRed2-N1 was used as the internal control. The intensities of both EGFP and RFP fluorescence were detected using an F-4500 fluorescence spectrophotometer (Hitachi, Tokyo, Japan). All of the experiments were repeated three times.

### Colony formation assay

Transfected HepG2 and SMMC-7721 cells were seeded into 12-well plates at 300 cells and 800 cells per well. After 7 or 14 days, when more than 50 proliferating cells had turned into a colony, the colonies were stained with crystal violet to observe and count the cells. The colony formation rate (colony number)/(seeded cell number) was determined.

### Flow cytometric analysis

At 24 h post-transfection, HepG2 and SMMC-7721 cells were seeded into 6-well plates in complete culture medium for growth and proliferation assay. The medium was then exchanged and the cells were cultured under serum-starved conditions for 48 h. The cells were then returned to complete medium and cultured for 24 h. Cells were re-suspended in 95 % ethanol after centrifugation, incubated at −20 °C overnight, washed with PBS and suspended in a solution containing propidium iodide (PI) staining buffer (PBS, 50 μg/ml PI, 0.1 mg/ml DNase-free RNase) for 30 min on ice. Cells were analyzed using a FACS Calibur flow cytometer (DB Biosciences) and FlowJo software (DB Biosciences).

### Cell migration assay

The migratory ability of HepG2 and SMMC-7721 cells was measured using transwell migration assays. After 24 h of transfection, cells were placed in 200 μl of RPMI-1640 or MEM-α without FBS and seeded in Transwell chambers (pore size, 8 μm; Corning Costar, Cambridge, MA, USA), which were placed into 24-well culture plates that were loaded with 20 % FBS RPMI 1640 or MEM-α (600 μl). The cells were cultured in 37 °C humidified incubator for 24 h, and the chambers were removed, fixed and stained after the upper surface was wiped to remove the non-migrated cells. Images were captured using a NISElements F 3.0 System (Nikon, Tokyo, Japan). Five random visual fields per transwell were analyzed for calculations. Each assay was performed three times.

## Results

### HBx promotes transcription of pri-miR-1269b via activating NF-κB

**Fig. 1:**
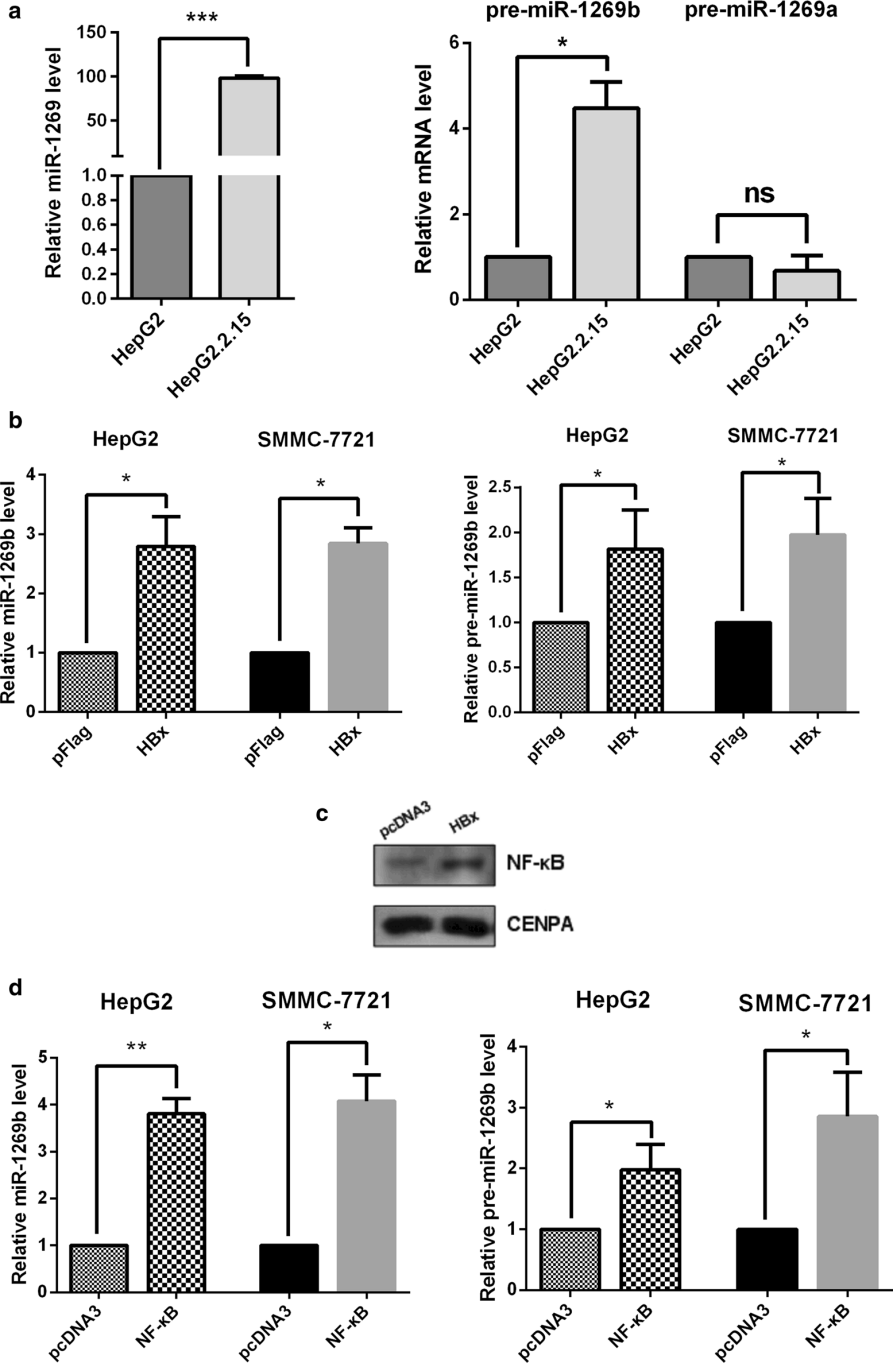
The level of miR-1269b was increased in HBV-HCC cells by pre-miR-1269b, which was up-regulated by HBx-activated NF-κB. **a** The relative expression levels of miR-1269b, pre-miR-1269b and pre-miR-1269a in HepG2 and HepG2.2.15 cells were detected using real-time PCR. **b** The relative expression level of miR-1269b in HepG2 and SMMC-7721 cells transfected with pFLAG or pFLAG-HBx. **c** HBx promoted the nuclear translocation of NF-κB nucleus, as confirmed by western blot analysis. **d** The relative levels of miR-1269b and pri-miR-1269b were up-regulated in cells transfected with NF-κB compared to their levels in cells that were transfected with the control vector. All *error bars* indicate the mean standard deviation based on three independent experiments. *p < 0.05, **p < 0.01

To explore whether the upregulation of miR-1269 in HBV-related HCC was derived from miR-1269a or miR-1269b, we performed RT-qPCR assay. Our data showed that the expression level of miR-1269 in HBV-produced cell line HepG2.2.15 is higher than that of HepG2 cells with HBV negative, and pre-miR-1269b level was significantly higher than pre-miR-1269a level in HepG2.2.15 cells compared with HepG2 cells (Fig. 1a), which suggest miR-1269 was induced by HBV. To determine whether HBx protein participates in this process, we ectopic expressed HBx in HBV-negative HCC cells. As shown in Fig. 1b, ectopic expression of HBx in HBV-negative HCC cells (HepG2 and SMMC-7721) increased the expression levels of pre-miR-1269b and miR-1269b. Since HBx protein does not bind to DNA sequence, a transcription factor that exactly controlled the expression of miR-1269b was taken into consideration. HBx can be a transcription activator of many host factors and NF-κB could be notably activated through the relocation from cytoplasm into nucleus by interacting with HBx (29). We detected nuclear NF-κB in HepG2 with HBx plasmid transfection, NF-κB content was evidently increased compared with the control vector (Fig. 1c). Meanwhile, overexpression of NF-κB induced miR-1269b and pre-miR-1269b expression in HepG2 and SMMC-7721 cells (Fig. 1d). Altogether, these results suggested that the higher expression of miR-1269b in HBV positive HCC cells may be caused by NF-κB which strongly activated by HBx protein.

### NF-κB binds to the promoter of miR-1269b to activate its expression

**Fig. 2:**
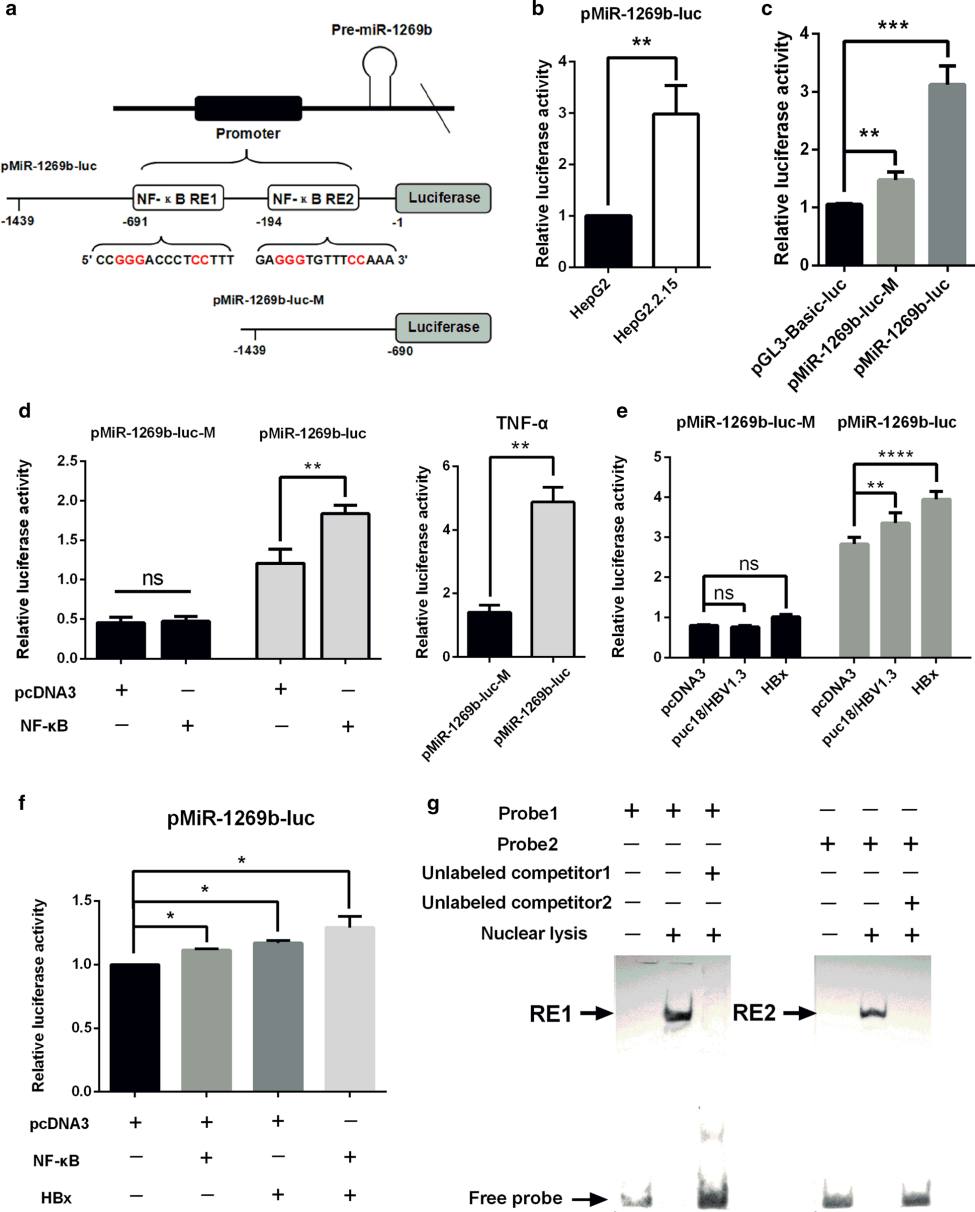
NF-κB binds directly to the miR-1269b promoter and up-regulates its transcription. **a** The human miR-1269b genomic locus. The predicted promoter of miR-1269b, which contains two putative binding sites for NF-κB (pMiR-1269b-luc), and the mutant of miR-1269b promoter that does not contain NF-κB binding sites (pMiR-1269b-luc-M) are shown. **b** miR-1269b promoter-induced luciferase activity was increased in HepG2.2.15 cells compared to HepG2 cells. **c** The relative luciferase activity induced by the miR-1269b promoters constructed with or without NF-κB binding sites and the control vector in SMMC-7721 cells. **d** The effect of NF-κB (*left*) and TNF-α (*right*) on pMiR-1269b-luc and pMiR-1269b-luc-M in SMMC-7721 cells. **e** SMMC-7721 cells were co-transfected with pMiR-1269b-luc and pMiR-1269b-luc-M with puc18/HBV1.3, pFLAG/HBx(HBx) and the control vector, and luciferase activity was then detected after 24 h of transfection. **f** SMMC-7721 cells were cotransfected with pMiR-1269b-luc and pcDNA3 or HBx or NF-κB or both HBx and NF-κB. After 24 h, luciferase activity was analyzed. **g** EMSA to determine the DNA-binding activity of NF-κB using two substrates (probe 1 and probe 2). The results are shown as the mean ± SD of three experiments that were performed in duplicate. *p < 0.05, **p < 0.01

To determine whether NF-κB promotes transcription of miR-1269b, we predicted the promoter of miR-1269b by utilizing bioinformatics website Promoter 2.0 Prediction Server (http://www.cbs.dtu.dk) and Promoter Scan (http://www-bimas.cit.nih.gov). The miR-1269b promoter was cloned in pGL3-basic vector (pMiR-1269b-luc) (Fig. 2a). Bioinformatic analysis indicated that miR-1269b promoter contains two binding sites of NF-κB (5′-GGGRNYYYCC-3′) (http://www.genomatix.de) (Fig. 2a). Luciferase reporter assay showed that luciferase activity in HepG2.2.15 cells was significantly higher than that in HepG2 cells (Fig. 2b). We constructed a mutant promoter plasmid (pMiR-1269b-luc-M) that deleted the region within NF-κB binding sites. As shown in Fig. 2c, pMiR-1269b-luc-M still possessed activity but dramatically decreased compared with pMiR-1269b-luc in non-NF-κB-activated SMMC-7721 cells. Next, overexpression of NF-κB or activation of NF-κB by low concentration of TNF-alpha (TNF-α) increased the pMiR-1269b-luc activity, but not affect the pMiR-1269b-luc-M activity in SMMC-7721 cells (Fig. 2d). To determine the effect of HBx on the promoter activity of miR-1269b, pMiR-1269b-luc and HBx or HBV expression plasmid, pHBV1.3 containing 1.3 copy of HBV genome in pUC18) [26] were co-transfected into HBV-negative HCC cells. Both HBx and pHBV1.3 plasmid induced miR-1269b promoter activity, but didn’t affect the activity of miR-1269b promoter mutant (Fig. 2e). Furthermore, luciferase reporter assay also demonstrated that HBx-induced miR-1269b expression was enhanced by overexpression NF-κB (Fig. 2f). To verify the direct interaction between NF-κB and miR-1269b promoter, EMSA assay was performed using biotin-labeled NF-κB consensus oligonucleotides in the miR-1269b promoter (−691 to −681) as probe 1, and miR-1269b promoter (−194 to −184) as probe 2. Nuclear extracts were incubated with probe1 or probe 2 or in the presence of two unlabeled, wild-type NF-κB binding probes. The wild-type NF-κB consensus oligonucleotides showed strong competition in combination with NF-κB (Fig. 2g). These results indicate that NF-κB directly activates the transcription of miR-1269b and HBx indirectly activates the transcription of miR-1269b in NF-κB dependent manner.

### miR-1269b promotes proliferation, cell cycle and migration in HCC cell lines

**Fig. 3:**
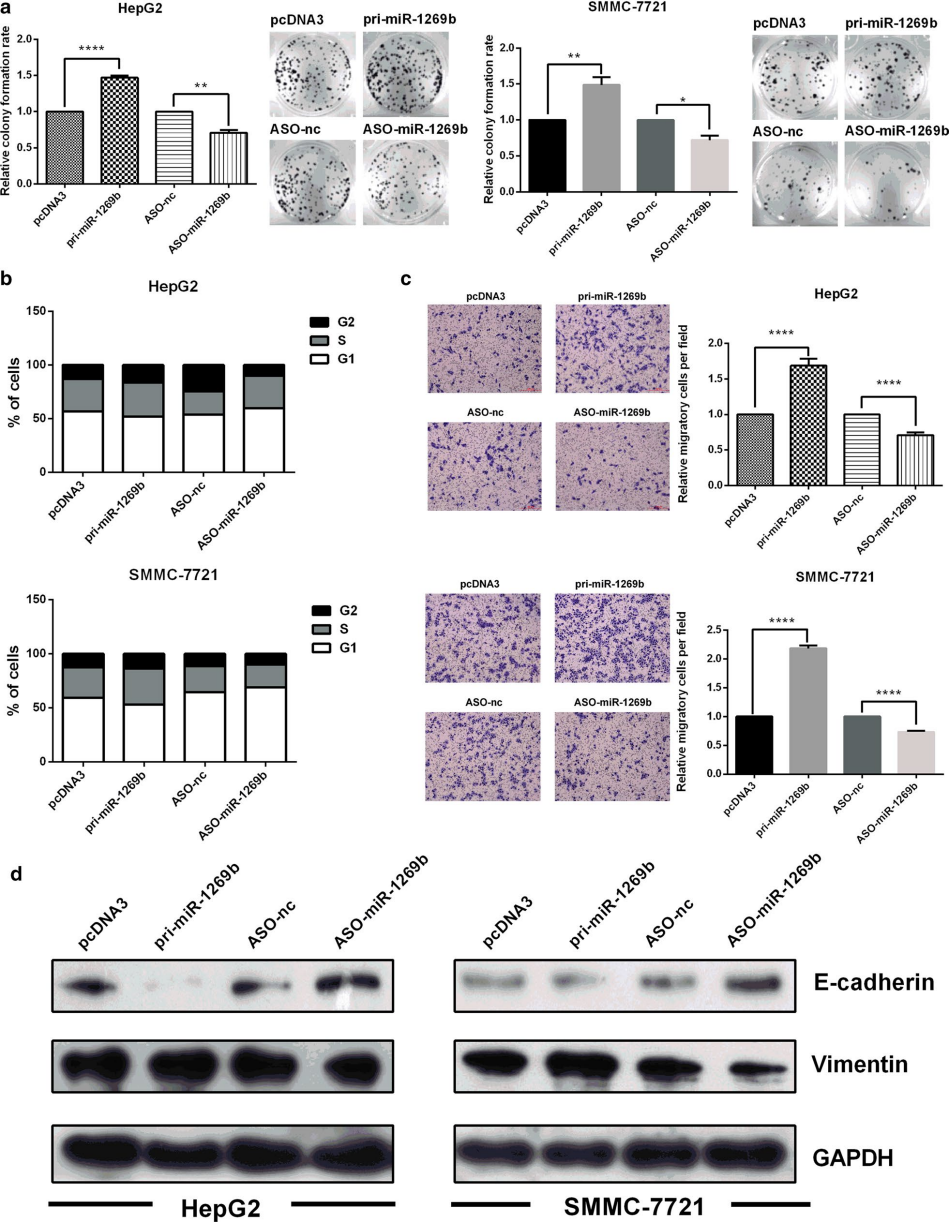
High expression levels of miR-1269b promoted the proliferation and migration of HepG2 and SMMC-7721 cells in vitro. **a** HepG2 and SMMC-7721 cells were transfected with pcDNA3/pri-miR-1269b, ASOmiR-1269b or a negative control vector in 24-well plates and then seeded in 12-well plates. The number of colonies was counted at 2 weeks after seeding. **b** Cell cycle progression was analyzed in HepG2 and SMMC-7721 cells using flow cytometry. The *chart* shows the population of cells that were in G1-, S- and G2/M-phase. **c** Transwell migration assays were performed to detect the migratory capacity of HepG2 and SMMC-7721 cells transfected with the same vectors. **d** The influence of miR-1269b on the protein levels of the EMT-associated molecules E-cadherin and vimentin were measured using western blot analysis. All *error bars* indicate the means ± SDs. All experiments were repeated at least three times. *p < 0.05, **p < 0.01, ***p < 0.001, ****p < 0.0001

The proliferation potential of miR-1269b was analyzed in HepG2 and SMMC-7721 cells transfected with miR-1269b overexpression (pri-miR-1269b) and miR-1269b antisense oligonucleotides (ASO-miR-1269b). Colony formation assay showed that overexpression of miR-1269b significantly promoted, but ASO-miR-1269b repressed cell proliferation in HepG2 and SMMC-7721 cells (Fig. 3a). Cell cycle analyses demonstrated that pri-miR-1269b enhanced, whereas ASO-miR-1269b delayed the transition of G1 to S/M phase (Fig. 3b). Transwell assay was applied to evaluate the effects of miR-1269b on migration in HCC cells. Cell migration was significantly enhanced in HepG2 and SMMC-7721 cells transfected with miR-1269b, while repressed in the cells transfected with ASO-miR-1269b compared to their respective control groups (Fig. 3c). The increase of cell migration ability was usually associated with EMT. The main protein markers of EMT including E-cadherin and vimentin were examined by western blot. As shown in Fig. 3d, overexpression of miR-1269b decreased E-cadherin and increased vimentin level, but ASO-miR-1269b resulted in an opposite effect, which was corresponded to phenotypic changes (Fig. 3d). Taken together, these results indicate that miR-1269b enhances oncogenic activity in HCC cells.

### miR-1269b enhances CDC40 expression by binding its 3′UTR in HCC cell lines

**Fig. 4:**
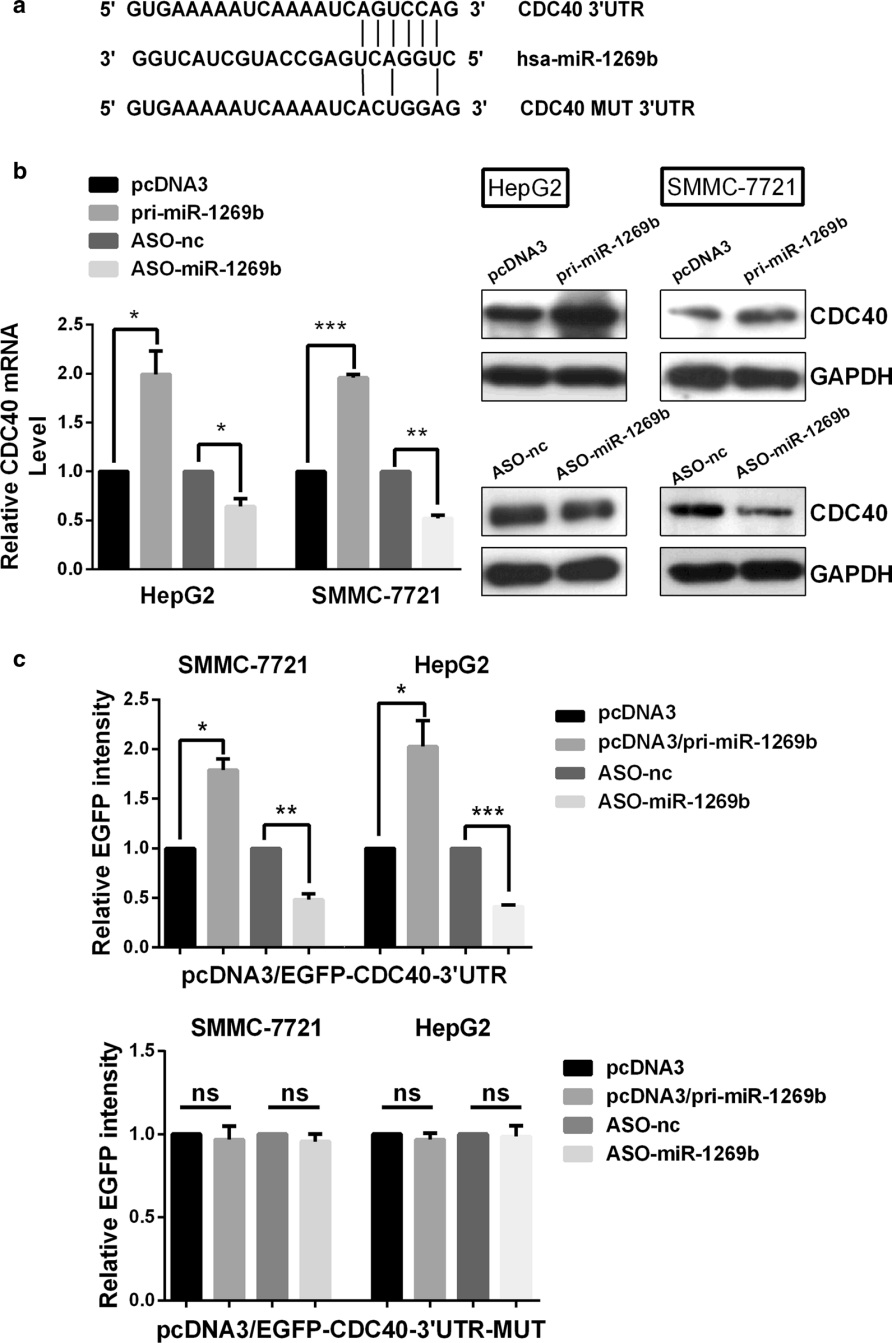
miR-1269b directly targets the 3′UTR of CDC40. **a** The predicted binding sites for miR-1269b in the 3′UTR of CDC40 and the induced mutations in the binding sites are shown. **b** qRT–PCR (*left*) and representative Western blot analysis (*right*) of CDC40 levels in HepG2 and SMMC-7721 cells transfected with the control vector or ASO-nc vs miR-1269b or ASO-miR1269b-transduced cells. **c** Luciferase reporter assays were performed in HepG2 and SMMC-7721 cell lines that were co-transfected with miR-1269b in the presence of CDC40 3′UTR (*top*). In the controls, the mutated 3′UTR sequences were included (*bottom*). Columns, mean ± SD of at least three independent experiments. *p < 0.05, **p < 0.01, ***p < 0.001

miRNAs generally functions as a regulator of gene expression by binding to the mRNA 3′UTR. Therefore we searched the potential target genes of miR-1269b using bioinformatics algorithms including TargetScan and miRBase Targets. Finally we chose CDC40 as a candidate target of miR-1269b because it regulates cell cycle progress and its impact in cancer cells was unclear. The miR-1269b binding site in the CDC40 mRNA 3′UTR is shown in Fig. 4a. To establish regulative relations between miR-1269b and CDC40, RT-qPCR and western blot assay were applied. As shown in Fig. 4b, it is surprised that CDC40 mRNA and protein expression level were up-regulated by overexpression of miR-1269b but down-regulated when miR-1269b was blocked by ASO in both HepG2 and SMMC-7721 cells. In addition, EGFP reporter assay also showed that overexpression of miR-1269b increased, but ASO-miR-1269b decreased fluorescence intensities, however, the mutated form (Fig. 4a) of 3′UTR binding site in CDC40 mRNA abolished the change of fluorescence (Fig. 4c). The results indicate that miR-1269b directly targets CDC40 and enhanced CDC40 expression in HCC cells.

### CDC40 promotes proliferation, cell cycle and migration and mediates promotion of malignancy induced by miR-1269b in HCC cell lines

**Fig. 5:**
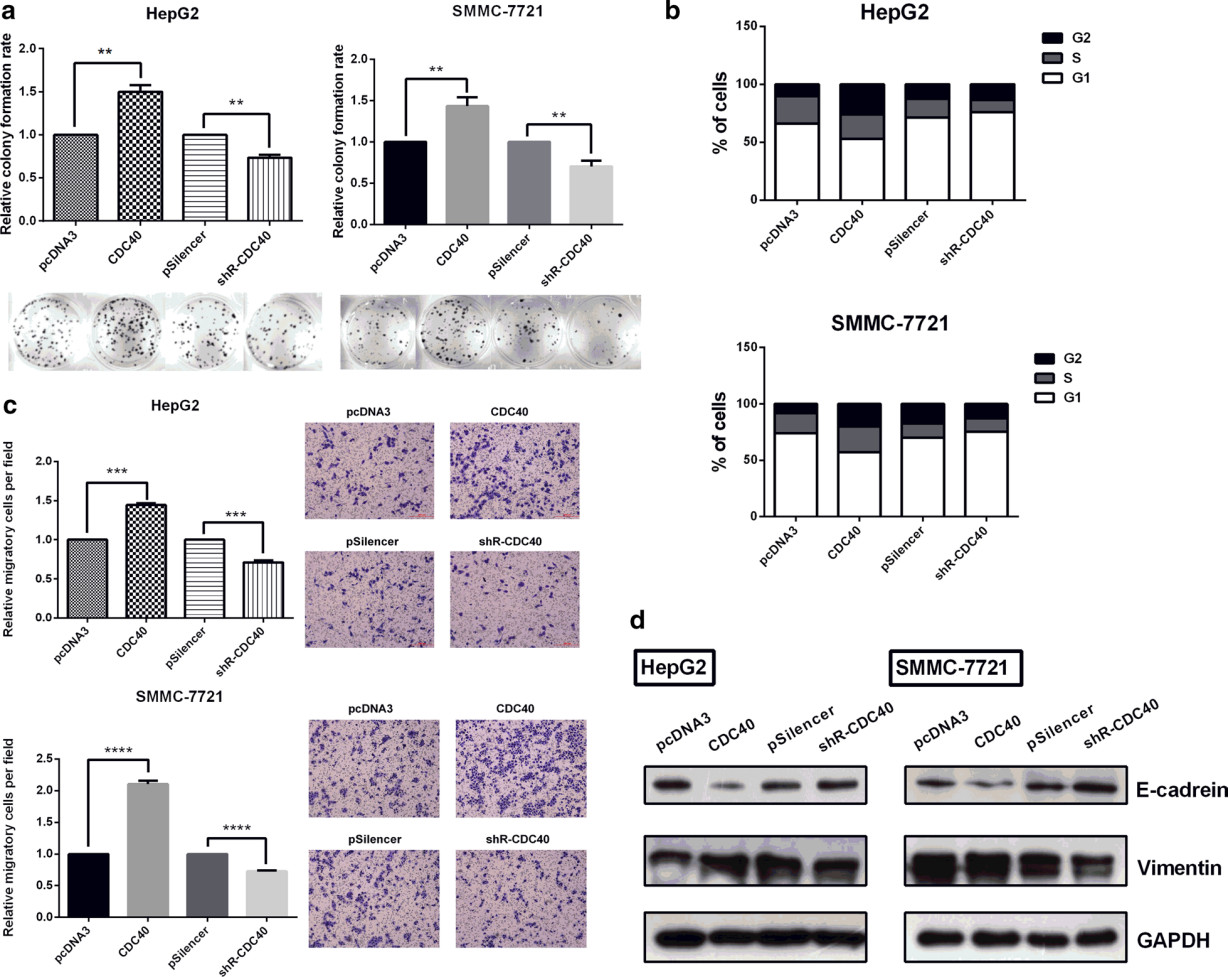
CDC40 promotes cell proliferation and migration, which is regulated by miR-1269b. **a** Colony formation assays were performed to test the influence of CDC40 on proliferation in HepG2 and SMMC-7721 cells. **b** Cell cycle progression in the transfected cells was analyzed using flow cytometry. The *chart* shows the populations of cells in the different phases of the cell cycle. **c** Transwell migration assays were performed to detect the effect of CDC40 on the migration of HepG2 and SMMC-7721 cells. **d** The influence of CDC40 on the protein levels of the EMT-associated molecules E-cadherin and vimentin was determined using western blot analysis. Cells were transfected 48 h before proteins were harvested. *Error bars* indicate the mean ± SD. *p < 0.05, **p < 0.01, ***p < 0.001

Cell proliferation and transwell assays were applied to elucidate the effects of CDC40 on of HCC cells. As shown in Fig. 5a, b, colony formation and cell cycle assays showed that CDC40 overexpression significantly enhanced, while knockdown of CDC40 by shRNA suppressed the growth and proliferation capacity in HepG2 and SMMC-7721 cells. Meanwhile, transwell assay showed that overexpression of CDC40 possessed stronger influence on migration, which can be impaired by reducing expression of CDC40 in HepG2 and SMMC-7721 cells (Fig. 5c). CDC40 also promoted EMT, which was consistent with change of the essential EMT protein markers. As shown in Fig. 5d, CDC40 overexpression promoted, but knock down of CDC40 repressed vimentin levels, but E-cadherin expression levels had oppositely changed by western blot assay. These results indicate that CDC40 promotes the tumorigenic activities in HCC cells.

**Fig. 6:**
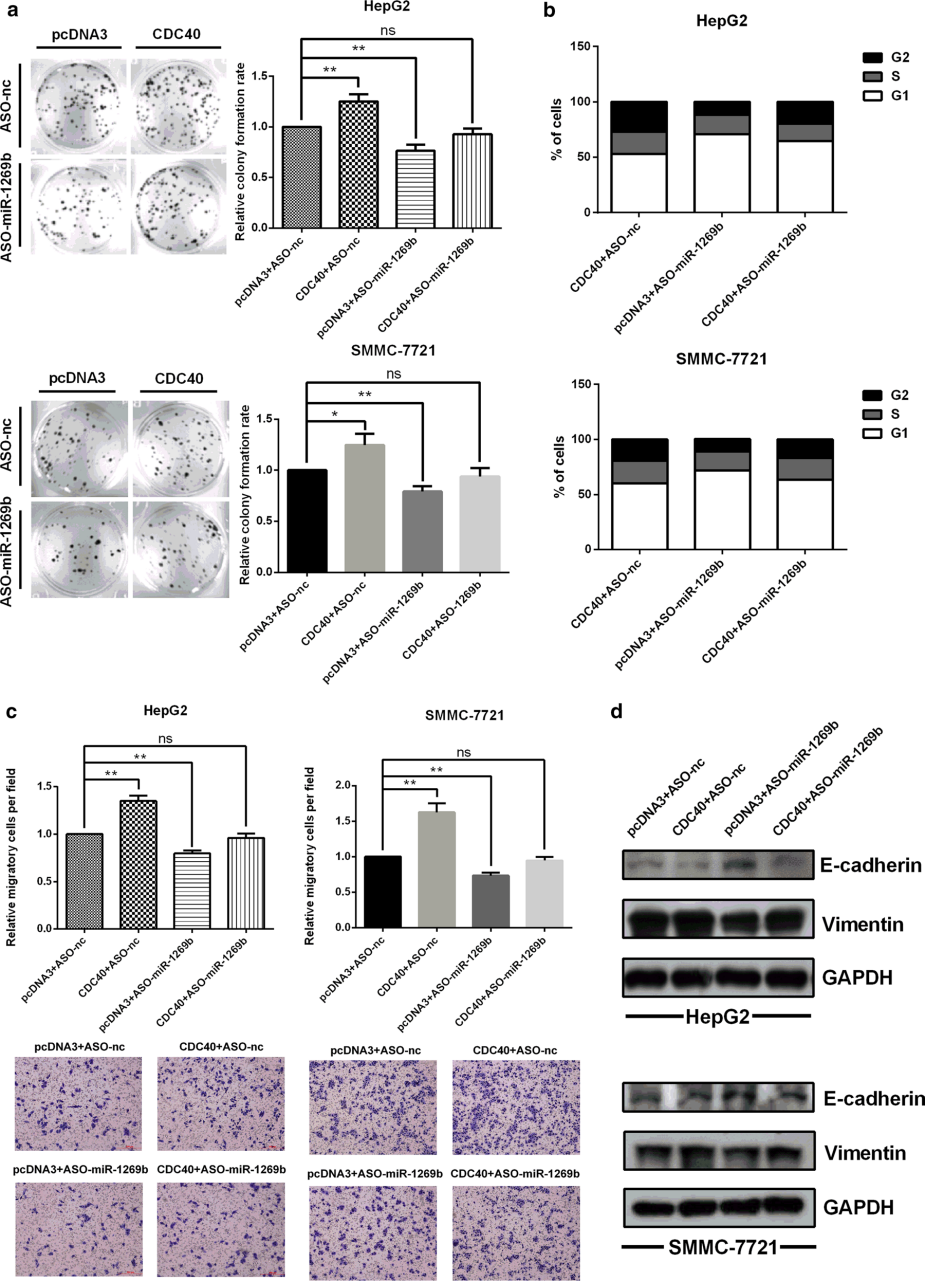
Overexpression of CDC40 abrogated the repression of the phenotypes induced by ASO-miR-1269b in HepG2 and SMMC-7721 cells. **a** Colony formation rates. **b** Cell cycle progression. **c** Migratory ability. **d** Corresponding levels of the EMT protein markers E-cadherin and vimentin. *Error bars* indicate the mean ± SD. *p < 0.05, **p < 0.01, ***p < 0.001

We have evidenced that miR-1269b up-regulates CDC40 expression and both CDC40 and miR-1269b possess the similar effect on malignancy in HCC cells. Thus it is necessary to verify the effect of miR-1269b on proliferation and migration was mediated by up-regulating CDC40 expression in HCC cells. Rescue experiments were performed in following approaches. Colony formation and cell cycle assays indicated that overexpressing CDC40 could reverse the inhibitory effect of the ASO-miR-1269b on proliferation in HepG2 and SMMC-7721 cells (Fig. 6a, b). Transwell migration analysis showed that the promoting effects of CDC40 on migration were abrogated when ASO-miR-1269b was co-transfected (Fig. 6c). Meanwhile, changes of EMT protein markers induced by miR-1269b were reversed by modulation of CDC40 expression levels in HepG2 and SMMC-7721 cells (Fig. 6d). Taken together, these results indicate that CDC40 promotes HCC cells proliferation and migration, and mediated the oncogenic activities induced by miR-1269b in HCC cells.

## Discussion

MiR-1269 is significantly up-regulated in HCC tissues and cell lines and promotes HCC growth by suppressing the FOXO1 gene [21, 22]. However, the mechanism of miR-1269b up-regulation has not been clarified. Because most HCC patients are HBV positive, we hypothesized that up-regulation of miR-1269 may be induced by HBV infection. As expected, our results indicated that miR-1269 was highly expressed in HBV-positive hepatoma HepG2.2.15 cells compared with the HBV-negative HepG2 and SMMC-7721 cells. Bioinformatics analysis showed that miR-1269 has two original transcripts, pre-miR-1269a located on chromosome 4 and pre-miR-1269b located on chromosome 17. To determine which precursor or whether both are induced by HBV infection, RT-qPCR was performed to detect the precursors of miR-1269a and miR-1269b. Our results indicated that miR-1269 and pre-miR-1269b, but not pre-miR-1269a, were highly expressed in HepG2.2.15 cells compared with the parental HepG2 cells. Additionally, to determine whether HBx activates the promoter of miR-1269b, we co-transfected a miR-1269b vector and HBx or HBV expression plasmid pHBV1.3 containing 1.3 copies of the HBV genome in pUC18 [26] into HBV-negative HCC cells. Overexpression of HBx induced miR-1269 and pre-miR-1269b in HBV-negative HepG2 and SMMC-7721 cells. Thus, we concluded that the HBV infection-induced expression of miR-1269 is due to the activation of miR-1269b expression.

Next, we determined how the HBV infection induced miR-1269b expression. The HBV X protein plays an important role in HBV-induced HCC, NF-κB could be significantly activated by HBx [27] and is involved in tumor progression due to its transcriptional regulation of various functional genes and miRNAs [8, 28]. Here, we revealed that the HBx protein promoted miR-1269b expression. Previous reports have shown that the HBx protein activates NF-κB by facilitating translocation of NF-κB from the cytoplasm into the nucleus [13–16]. Therefore, we hypothesized that the activation of NF-κB by the HBx protein may induce miR-1269 expression. Our data indicated that the promoter of miR-1269b was activated by HBx and NF-κB using luciferase reporter assays, and mutation of the predicated NF-κB binding sites in the miR-1269b promoter abolished the luciferase activity. EMSA assays confirmed the direct interactions between NF-κB and the predicted binding sites in the miR-1269b promoter region. Thus, we conclude that the HBx protein promotes miR-1269b expression in a NF-κB-dependent manner.

miR-1269b promoted cell proliferation, cell cycle and migration in HepG2 and SMMC-7721 cells and functioned as an oncogene in HCC cells. Because the effects of miRNAs are mediated by its targets, bioinformatics analysis identified CDC40 as a candidate target of miR-1269b. Using EGFP reporter assays, as well as RT-qPCR, western blot analysis and a series of rescue experiments, we confirmed that CDC40 was up-regulated by miR-1269b and a functional target of miR-1269b in HCC cells. CDC40 has been associated with the spliceosome, which regulates pre-mRNA splicing [29, 30], and was also linked to cell cycle control [23, 24]. CDC40 exhibits a delayed G1/S transition and mutated form causes cell cycle arrest at the G2/M stage [24, 31]. However, except for one report showing CDC40 as a target of miR-378 in colorectal cancer [32], the correlation between CDC40 and tumor malignancy has not been investigated.

In the present study, CDC40 was shown to enhance cell growth, cell cycle progression and migration in HepG2 and SMMC-7721 cells by gain- and loss-of-function analysis. Migration is generally associated with cancer metastasis, while EMT plays a crucial role in cancer metastasis in HCCs [33]. E-cadherin belongs to a family of transmembrane glycoproteins, which are responsible for calcium-dependent cell-to-cell adhesion and loss of E-cadherin contributes to the cell migration. Meanwhile, vimentin is a major protein of the intermediate filament family and has also been recognized as a marker for EMT. In EMT progression, the epithelial differentiation marker E-cadherin is down-regulated, and the mesenchymal marker vimentin is up-regulated. In our study, we showed that CDC40 could enhance the HCC cell migration via ablation of E-cadherin and enhancement of vimentin. Moreover, phenotypic rescue experiments demonstrated that CDC40 at least partially mediated the promotion of the oncogenic activities induced by miR-1269 in HCC cells. However, the mechanism by which miR-1269b up-regulates CDC40 expression needs to be elucidated.

## Conclusion

**Fig. 7:**
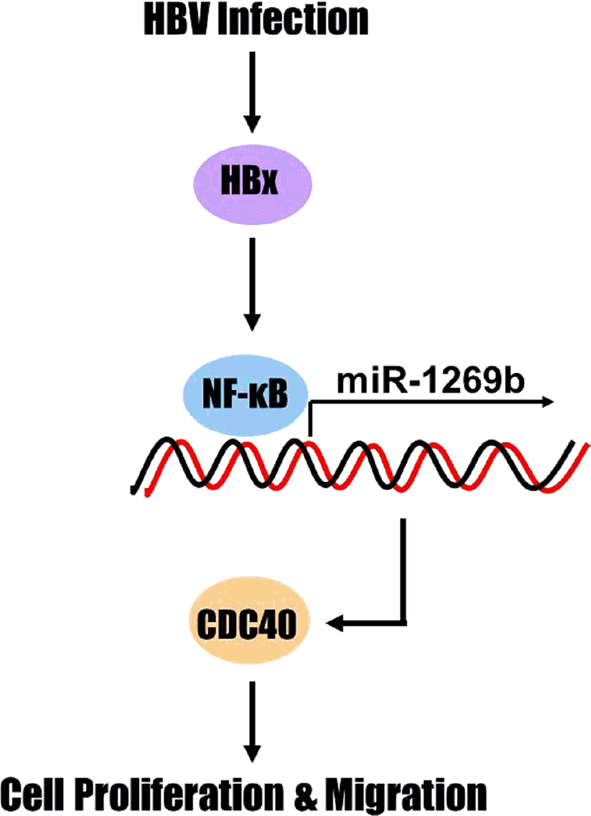
The signaling pathway by which HBx-induced miR-1269b facilitates proliferation and migration in HBV-positive HCC cells

Our finding revealed that HBx protein facilitates NF-κB to import into nucleus, resulting in activation of miR-1269b expression. Upregulation of miR-1269b enhances CDC40 protein level by binding its 3′UTR, which promotes the growth and migration in HCC cells. Thus, our results presented a novel pathway of HBx/NF-κB/miR-1269b/CDC40 (Fig. 7) in tumorigenesis in HBV-positive HCC and also may provide new potential biomarkers of clinical study in HCC.

## Abbreviations

CDC40: cell division cycle 40 homolog
EGFP: enhanced green fluorescent protein
EMSA: electrophoretic mobility shift assay
EMT: epithelial mesenchymal transitions
HBV: hepatitis B virus
HBx: HBV X protein
HCC: hepatocellular carcinoma
miRNAs: microRNAs
NF-κB: nuclear factor kappa-light-chain-enhancer of activated B cells
RT-qPCR: reverse transcription quantitative polymerase chain reaction
TNF-α: tumour necrosis factor alpha
